# Outcome and impact of Master of Public Health programs across six countries: education for change

**DOI:** 10.1186/1478-4491-12-40

**Published:** 2014-08-06

**Authors:** Prisca AC Zwanikken, Nguyen Thanh Huong, Xiao Hua Ying, Lucy Alexander, Marwa SE Abuzaid Wadidi, Laura Magaña-Valladares, Maria Cecilia Gonzalez-Robledo, Xu Qian, Nguyen Nhat Linh, Hanan Tahir, Jimmie Leppink, Albert Scherpbier

**Affiliations:** 1 Royal Tropical Institute, PO Box 95001, 1090 HA Amsterdam, The Netherlands; 2 Hanoi School of Public Health, 138 Giang Vo, Kim Ma, Ba Dinh, Hanoi, Vietnam; 3 School of Public Health, Fudan University, 138 Yixueyuan Road, Box 175, Shanghai 200032, PR China; 4 School of Public Health, University of the Western Cape, Private Bag X17, Bellville 7535, Republic of South Africa; 5 Human Resource Development, Federal Ministry of Health, PO Box 303, Khartoum, Sudan; 6 National Institute of Public Health, Universidad No. 655 Colonia Santa María Ahuacatitlán, Cerrada Los Pinos y Caminera, CP 62100 Cuernavaca, Morelos, México; 7 Research Centre in Health Systems, National Institute of Public Health, Universidad No. 655 Colonia Santa María Ahuacatitlán, Cerrada Los Pinos y Caminera, CP 62100 Cuernavaca, Morelos, México; 8 MPH Programme, University of Medical Sciences and Technology, PO Box 12810, Khartoum, Sudan; 9 Faculty of Health, Medicine and Life Sciences, Maastricht University, PO Box 616, 6200 MD Maastricht, The Netherlands

**Keywords:** Evaluation, Graduate, Impact, Low- and middle-income countries, Master of Public Health, Outcome

## Abstract

**Background:**

The human resources for health crisis has highlighted the need for high-level public health education to add specific capacities to the workforce. Recently, it was questioned whether Master of Public Health (MPH) training prepared graduates with competencies relevant to low- and middle-income countries (LMICs). This study aims to examine the influence of the MPH programs geared towards LMICs offered in Vietnam, China, South Africa, Mexico, Sudan, and the Netherlands on graduates’ careers, application of acquired competencies, performance at the workplace, and their professional contribution to society.

**Methods:**

A self-administered questionnaire was sent to graduates from six MPH programs. Frequency distributions of the answers were calculated, and a bivariate analysis and logistic regression of certain variables was performed.

**Results:**

The response rate was 37.5%. Graduates reported change in leadership (69%), in technical position (69%), acquiring new responsibilities (80%), and increased remuneration (63%); they asserted that MPH programs contributed significantly to this. Graduates’ attribution of their application of 7 key competencies ‘substantially to the MPH program’ ranged from 33% to 48%. Of the 26 impact variables, graduates attributed the effect they had on their workplace substantially to the MPH program; the highest rated variable ranged from 31% to 73% and the lowest ranged from 9% to 43%. Of the 10 impact variables on society, graduates attributed the effect they had on society substantially to the MPH program; for the highest rated variable (13% to 71%); for the lowest rated variable (4% to 42%). Candidates’ attribution of their application of acquired competencies as well as their impact at the workplace varied significantly according to institution of study and educational background.

**Conclusions:**

This study concludes that these MPH programs contribute to improving graduates’ careers and to building leadership in public health. The MPH programs contribute to graduates’ application of competencies. MPH programs contribute substantially towards impact variables on the workplace, such as development of research proposals and reporting on population health needs, and less substantially to their impact on society, such as contributing equitable access to quality services. Differences reported between MPH programs merit further study. The results can be used for curriculum reform.

## Background

The human resources for health crisis, i.e., the severe shortage of human resources in 57 low- and middle-income countries (LMICs), has highlighted the need for high-level public health education to add specific capacities to the workforce [1-5]. However, questions have been posed as to whether Master of Public Health (MPH) training prepared graduates with competencies relevant for LMICs [6-8]. These questions have also been raised in high-income countries [9,10] and were probably influenced by the general debate on the impact of higher education [11-15]. In addition, WHO identified evaluation of the education of health professionals as a knowledge gap [16].

Measuring outcome and impact of educational programs is fraught with methodological difficulties [14,15,17]. Blömeke pointed to the dearth of literature measuring competencies of students and graduates in higher education, especially internationally comparable measurements [15]. A systematic review of Master’s in Health and Health Care programs showed that outcome was usually measured through alumni surveys. In these alumni surveys, however, no questions on whether graduates attributed their advancement in their career or their application of competencies to the Master’s program were included. Other methods used, though less often, were focus group discussions, employer surveys, and semi- or unstructured interviews [18]. Furthermore, although alumni were sometimes asked what they accomplished in their work, these questions were open-ended and did not address outcome or impact indicators. Impact on the workplace was measured in four studies [19-22] and impact on society was only reported in two studies [20,22]. Impact was not measured in a systematic manner in any of these studies. Self-reported competency and academic outcome by students and graduates is a valid measure for higher education learning as shown by earlier studies [15,23-27].

Outcome in this study is defined as the application of competencies and as the effects on career, such as increase in leadership, new responsibilities, change in position, and increase in remuneration. Impact in this study is defined as impact on the workplace, e.g., “developed a study or a research proposal” and impact on sector or society, e.g., “contributed to equitable access to quality services”. Competencies for the MPH and impact variables on work and society were jointly constructed and validated prior to the study. The designed competencies were based on the competencies and learning objectives of the six participating institutions offering MPH programs and the set of competencies of the Council on Linkages Between Academia and Public Health Practice as a reference. The competencies and impact variables were validated with experts in the field and alumni in the six different countries [28].

The aim of this study was to analyze the influence of the MPH programs on graduates’ careers and their leadership, on application of competencies acquired in the MPH program as well as on impact at the workplace and on their contribution to society.

## Methods

This is a cross-sectional study of graduates from six MPH programs: Hanoi School of Public Health, Vietnam (HSPH), School of Public Health Fudan University, China (SPHFU), School of Public Health University of Western Cape, South Africa (SPHUWC), National Institute of Public Health Mexico, Mexico (INSP), University of Medical Sciences and Technology, Sudan (UMST), and the Royal Tropical Institute, Netherlands (KIT). All offer MPH programs geared towards LMICs. HSPH and KIT offer fulltime programs, SPHFU offers a part-time program and, since 2010, a fulltime program. At SPHUWC, INSP, and UMST students can follow the programs full- and part-time.

An anonymous self-administered questionnaire was designed, based on the analytical framework of the systematic review by Zwanikken and a previous questionnaire [18,29]. As attribution was rarely addressed in articles reviewed [18], specific questions were asked regarding the graduates’ attribution of competencies and impact variables to the MPH program. The range of ratings was kept small to avoid the recognized tendency for respondents to repeat a rating where the range is wider (Additional file 1) [24,26].

The questionnaire was pretested with graduates from different years in all countries and revised, based on comments received. In Vietnam, Mexico, and China the questionnaire was translated into the national language and translated back to check for consistency of the translation. The questionnaire was administered by each institution through free online tools or through email (Table 1). Graduates were reminded two times by email, or by telephone. The questionnaire targeted graduates from the MPH programs of the six participating institutions from 2005–2010, in total 1,187 graduates, to allow sufficient time for graduates to have applied their newly gained competencies. The questionnaire was online throughout November 2012 to February 2013.

**Table 1:** Approaching graduates and tools used

**Institution**	**How graduates were approached**	**Tool for filling in questionnaire**
HSPH (Vietnam)	By email/reminder by telephone	Questionnaire send through email
SPHFU (China)	By telephone	http://www.sojump.com
SPHUWC (South Africa)	By email	http://www.surveymonkey.com
INSP (Mexico)	By email	Webserver of the institute
UMST (Sudan)	By email/telephone	Questionnaire send through email or hard copy
KIT (The Netherlands)	By email	http://www.surveymonkey.com

Prior to embarking on the study, the ethics committees of the six participating institutions, i.e., the University of Western Cape Senate Research and Ethics Committee, Hanoi School of Public Health Ethics Committee, Fudan University School of Public Health Institutional Review Board, Sudan Medical and Scientific Research Institute Ethical Clearance Committee, National Institute of Public Health Mexico Ethics Committee, and the Royal Tropical Institute Research Ethics Committee, granted ethical approval for the study.

### Data analysis

The answers from all institutions were analyzed using Microsoft Excel 2013 and SPSS 21. Descriptive and bivariate analysis of specific variables was performed.

Logistic regression was performed to examine whether a medical doctor background (yes/no), additional degree (yes/no), institution, gender, time of graduation (2005–2007 or 2008–2010), and age (in years) can predict change in leadership level (yes/no), change in technical position (yes/no), change to position involving more responsibility (yes/no), increase in remuneration (yes/no), and/or a change to another employer (yes/no).

We performed analysis of variance (ANOVA) and analysis of covariance (ANCOVA) to examine which of the aforementioned predictor variables yield a significant contribution to the perceived extent to which MPH contributed to a change in leadership, technical position, employer, and/or increase in remuneration.

The questionnaire included a component on the extent to which MPH contributed to application of acquired competencies (Cronbach’s *α* = 0.957), to graduates’ performance at the work place (Cronbach’s *α* = 0.954), and to their contribution to society (Cronbach’s *α* = 0.940). For each of these three components, exploratory factor analysis using generalized least squares estimation was performed to compute factor scores following the Anderson-Rubin method [30]. These standardized factor scores, having a mean of zero and standard deviation of approximately one, were used as response variables in ANOVA and ANCOVA to examine which of the aforementioned predictor variables yield a significant contribution to a higher extent of application of acquired competencies, better performance of graduates at the workplace, and an increased impact on society. For all ANOVAs and ANCOVAs, Eta-squared (*η*^2^) was used as measure of effect size. Values of 0.01 indicate small effects, values of 0.06 indicate medium size effects, and values of 0.14 are indicative of large effects [30].

While Anderson-Rubin factor scores are generally somewhat more precise than simple sum or average scores, a drawback of these factor scores is that one cannot use them to assess the degree of contribution of the MPH program to each – application of competencies or workplace performance or impact on society – because the mean of the aforementioned factor scores is zero. Therefore, for the latter, a proportion (i.e., a value somewhere between 0 and 1) was calculated for each of these three components; we divided the number of “substantial attribution” responses by the number of items in the component. We then performed between-subjects-by-within-subjects ANOVA, treating the three components as within-subjects factors and treating institution as the between-subjects factor.

## Results

### Response rate and demographics of respondents

The overall response rate was 37.5%. The highest response rate was obtained in Vietnam, and the lowest in Mexico and China, but overall the response rate between the institutions did not differ much (Table 2).

**Table 2:** Response rate and demographics of respondents

	**Number of respondents**	**Response rate (%)**	**Female respondents (%)**	**Male respondents (%)**	**Median year born**	**Medical doctors (%)**
HSPH (Vietnam)	153	52	57	43	1971	64
KIT (The Netherlands)	86	39	43	57	1972	47
INSP (Mexico)	61	26	71	29	1972	56
SPHFU (China)	60	26	43	57	1975	22
SPHUWC (South Africa)	50	39	54	46	1967	22
UMST (Sudan)	35	41	48	52	1976	54
Total/Average	445	37.5	51	49	1972	49

Of the respondents, 50.8% were female and the median year of birth was 1973, ranging from 1955–1997. Respondents had professional educational backgrounds in medicine (48.5%), Bachelor of Public Health (9.9%), nursing (7.4%), dentistry (6.3%), social science (3.8%), nursing/midwifery (2.3%), pharmacy (2%), BSc or BA (5.4%), or other (14.3%). Most respondents (69%) studied fulltime. All graduates from HSPH and KIT studied fulltime; all alumni from SPHFU studied part-time. Graduates had an average work experience of 9.2 years (median 8 years) prior to the MPH, ranging from 0–30 years of work experience.

### Career and leadership

The effect on career and leadership were measured by changes in level of employment, leadership, technical position, responsibilities, remuneration, and graduates’ attribution to the MPH.

#### Level of employment before MPH and currently

**Figure 1:**
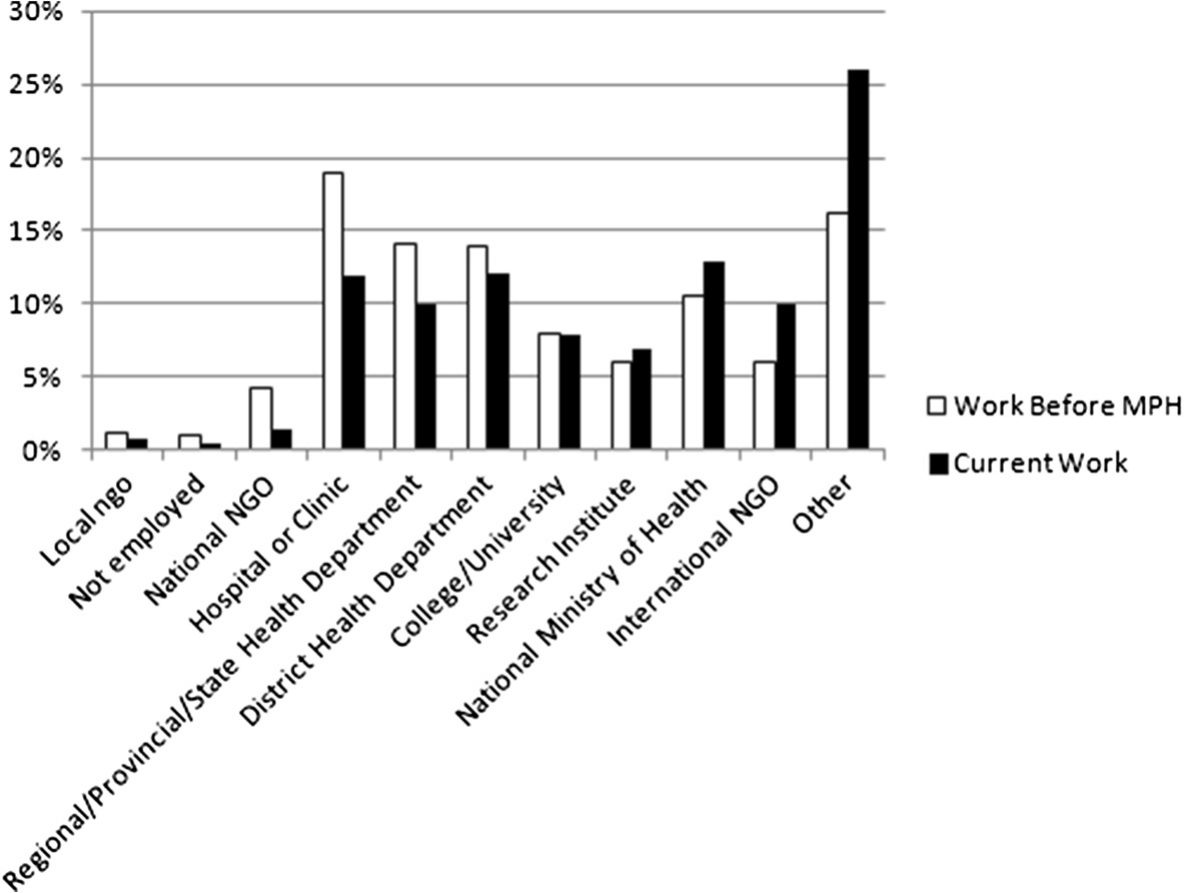
Level of employment of graduates before MPH and currently (n = 445).

Almost 50% of the alumni indicated that they worked in a clinic (18.9%) or at district (13.9%) or state health public health service (14.1%) prior to the MPH. After graduation, more than 50% of the graduates shifted towards working for the national Ministry of Health (12.7%), international non-governmental organizations (9.9%), a research institute (7%), or other (26%) (Figure 1).

Only 5% [24] of the graduates reported working currently outside their home country; this included two graduates who originated from a high income country. Fifteen of the graduates working outside their home country worked within the region (i.e., Africa), while seven of them went to work in a high income country. No graduates from the schools of China and Vietnam worked outside their country, and only two from Mexico did so.

#### Changes in leadership, technical position, responsibilities, and remuneration

**Figure 2:**
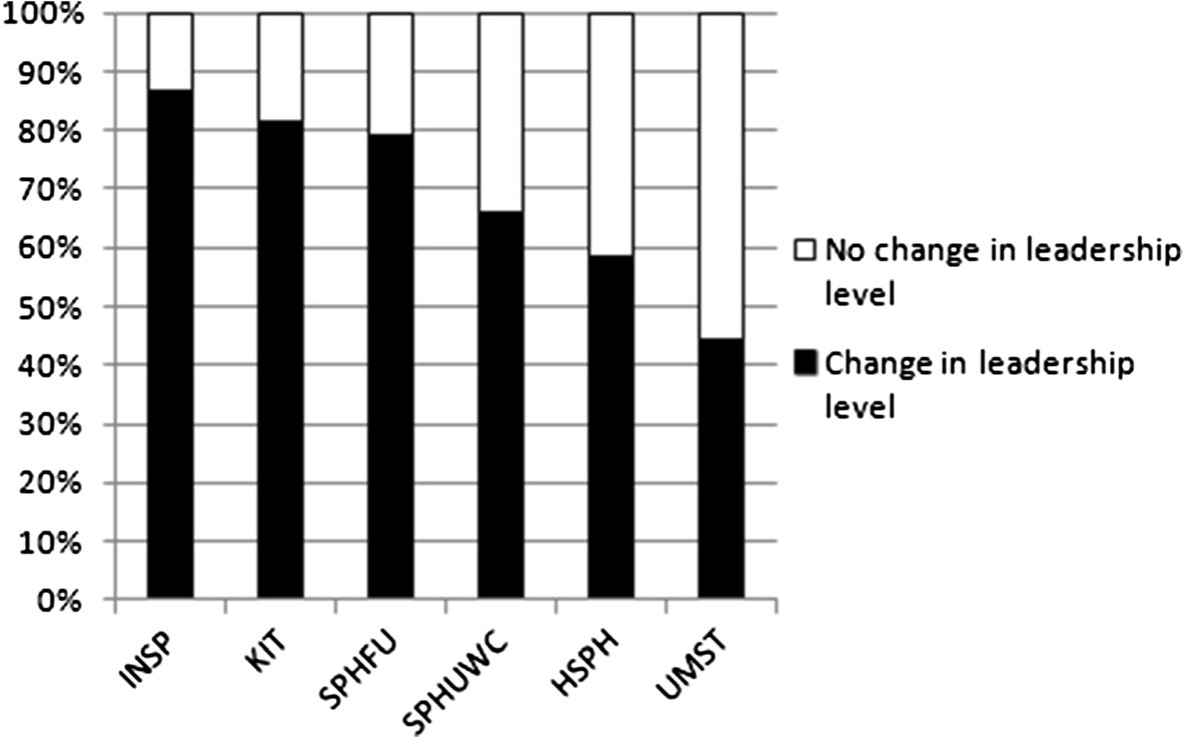
Reported change in leadership level by graduates, % per school, n = 419*. *Missing: 26.

Graduates reported a change in leadership in the management system after the MPH (mean: 69%, for ranges see Figure 2), a change in technical position or area of focus (mean: 69%; range: 57% to 85%), acquisition of new responsibilities (mean: 80%; range: 53% to 100%). More than half of the graduates (mean: 63%; range: 53% to 81%) reported an increase in remuneration, while 32.7% remained the same.

Graduates were asked to attribute the change in leadership, technical position, remuneration, and change of employer to the MPH graduation on a scale of 1–5 (insignificant to very significant). According to graduates, the MPH program contributed substantially to a change in leadership: nearly 76% responded this to be significant or very significant; as not enough people reported no change in leadership, a correlation could not be computed. The MPH program also contributed to change in employer: about 65% responded this to be significant or very significant. Furthermore, the MPH program was reported to contribute to change in technical position (*ρ* = 0.371, *P* <0.001) and to increase in remuneration (*ρ* = 0.430, *P* <0.001).

#### Further training after the MPH

More than half of the graduates (57%) reported having completed certified work-related training of 2 weeks or more, with a range from 1 to more than 10 courses. About a third (34%) took a further degree or diploma, other than short training courses, after the MPH. Graduates reported to have taken a PhD (9%), diploma (9%), another Master degree (7%), a postgraduate diploma (1%), or other (10%). Graduates were eager to pursue further studies: 74% planned another degree.

### The extent to which the MPH program enabled the graduate to apply specific public health competencies in their work

Seven core competencies were subdivided into detailed competencies. Graduates were asked to grade attribution to the MPH program per detailed competency (23 in total), scaled as follows: they did not use it/it was not part of their work; the MPH program did not enable the graduate; the MPH program enabled the graduate a little to apply; or enabled the graduate substantially to apply this competency in their work. Graduates stated that the MPH program enabled them substantially to apply the following core competencies: public health science skills including analytical assessment competencies (48%), leadership and systems thinking competencies (44%), context sensitive competencies (43%), and planning and management competencies (42%). About a third of the graduates stated that the MPH program enabled them substantially to apply the three following core competencies: communication competencies (37%), community and inter-sectoral competencies (36%), and policy development competencies (33%). Strikingly, within all competencies, there were graduates that did not use the competency or it was not part of their work, with a range from 9 to 19%, of which the highest was the policy development competency (19%) (Table 3).

**Table 3:** Enablement of application of specific public health competencies attributed to the MPH program as reported by graduates (n = 420)*

Public health competencies/Attribution to MPH program*	**MPH enabled me substantially to apply**	**MPH enabled me a little to apply**	**Not due to MPH**	**Not used/not part of my work**
Public health science skills including analytical assessment competencies	48%	34%	9%	9%
Leadership and systems thinking competencies	44%	36%	10%	11%
Context sensitive competencies	43%	33%	12%	12%
Planning and management competencies	42%	33%	9%	15%
Communication competencies	37%	39%	13%	11%
Community and inter-sectoral competencies	36%	34%	16%	15%
Policy development competencies	33%	36%	12%	19%

*Missing: 25.

### The extent to which the MPH program enabled the graduate to impact on the workplace

Graduates were asked to attribute their impact on the workplace to the MPH program as follows: they did not use it/it was not part of their work; the MPH program did not enable the graduate; the MPH program enabled the graduate a little; or enabled the graduate substantially to impact on workplace. Graduates stating that the MPH program enabled them substantially to impact on their workplace ranged from 60% to 20% for the 26 variables. The three highest scored variables were “developed a study or a research proposal” (mean 60%; range: 73% to 32%), “reported and made recommendations on population health status or needs” (mean 43%; range: 61% to 31%), and “made presentations at conferences” (mean 41%; range: 68% to 21%). The three lowest scored variables were “published or posted in popular (including electronic) media” (mean: 23%), “contributed to change in policy at one level higher than work institution” (mean: 22%), and “contributed to writing a published chapter of a book” (mean: 20%) (Additional file 2).

### The extent to which the MPH program enabled the graduate to impact on society

Graduates were asked to grade whether the MPH program enabled them to impact on society as follows: they did not use it/it was not part of their work; the MPH program did not enable the graduate; the MPH program enabled the graduate a little to impact; or enabled the graduate substantially to impact on society. Graduates stated that the MPH program enabled them substantially to impact on society with a range from 39% to 17% for the 10 variables. The three highest scored variables were “influenced better understanding of public health measures amongst general population” (mean: 39%; range: 71% to 13%), “contributed to equitable access to quality services” (mean 32%: range: 60% to 12%), and “contributed to increased resource mobilization for disadvantaged groups” (mean: 31%). The three lowest scored variables were “contributed to changes in policy or strategy in general” (mean: 25%), “contributed to equity/pro-poor orientation towards health access at all levels” (mean: 25%), and “contributed to changed guidelines, regulations, ordinances beyond the workplace” (mean: 17%) (Additional file 3).

### Changes in position, competencies, and impact variables by predictor variable

Graduates with a medical doctor background or other additional degree appear to be more likely to change leadership after graduation (*P* <0.001). Candidates graduating from HSPH, SPHUWC, or UMST appear to be less likely to change leadership than graduates from other institutes (Additional file 4: Table S6). Graduates with an additional degree appear to be somewhat more likely to change technical position. Graduates from HSPH, SPHFU, or UMST appear to be less likely to change technical position than graduates from other institutes (Additional file 4: Table S7). Graduates with an additional degree appear to be more likely to switch to a position involving more responsibility and graduates from INSP and KIT appear to be more likely to switch to a position involving more responsibility than graduates from other institutes (Additional file 4: Table S8).

Not unexpectedly, respondents who graduated fairly recently are less likely to have had an increase in remuneration; on average, they have been on the job market for less time than candidates who graduated before 2008. Men appear to be more likely to have an increase in remuneration than women (*P* = 0.005), and graduates from HSPH, SPHFU, or UMST appear to be somewhat less likely to have an increase in remuneration (Additional file 4: Table S9).

Recent graduates appear to be less likely to have switched employer than respondents who graduated before 2008. Furthermore, graduates from HPSH and SPHFU appear to be somewhat less likely to switch to another employer than graduates from other institutes (Additional file 4: Table S10).

Institutional differences were found with regard to MPH contribution to change in leadership, *F*(5, 303) = 16.217, *P* <0.001, *η*^2^ = 0.211; change in technical position, *F*(5, 303) = 19.762, *P* <0.001, *η*^2^ = 0.237; increase in remuneration, *F*(5, 303) = 15.822, *P* <0.001, *η*^2^ = 0.196; and a change in employer, *F*(5, 303) = 9.983, *P* <0.001, *η*^2^ = 0.182. With regard to contribution to change in leadership and contribution to change in technical position, graduates from KIT, INSP, and SPHUWC gave significantly higher responses than did graduates from HSPH, SPHFU, and UMST. With regard to MPH contributing to change in remuneration, graduates from KIT and SPHUWC gave significantly higher responses than did graduates from the other four institutions. Finally, with regard to attribution to change in employer, graduates from KIT, SPHUWC, and UMST gave higher responses than respondents from HSPH, SPHFU, and INSP. No other statistically significant predictor variables were found.

Institution also contributed significantly to differences between candidates in reported application of acquired competencies, *F*(5, 397) = 13.178, *P* <0.001, *η*^2^ = 0.166, with graduates from KIT, INSP, and SPHUWC responding significantly higher than graduates from HSPH, SPHFU, and UMST. Besides, graduates with a medical doctor background responded significantly higher than graduates without such a background, *F*(1, 397) = 8.931, *P* = 0.003, *η*^2^ = 0.022. The same group of institutions (KIT, INSP, and SPHUWC), *F*(5, 376) = 14.286, *P* <0.001, *η*^2^ = 0.160, and medical doctor background, *F*(1, 376) = 10.278, *P* = 0.001, *η*^2^ = 0.027, significantly contributed to differences in performance at the workplace, with graduates from SPHUWC, INSP, and KIT giving significantly higher ratings than graduates from HSPH, SPHFU, and UMST.

Institution where graduates studied also explained part of the differences between candidates in increased impact on society, *F*(5, 403) = 11.435, *P* <0.001, *η*^2^ = 0.124, with graduates from KIT and SPHUWC responding somewhat higher than graduates from other institutions. Furthermore, graduates with an additional degree rated impact higher than graduates without an additional degree, *F*(1, 403) = 4.681, *P* = 0.031, *η*^2^ = 0.011.

Finally, split-plot ANOVA suggests that, for all institutions together, the MPH program contributed slightly more to application of competencies to graduates’ performance at the workplace than to their contribution to society, *F*(1, 373) = 13.863, *P* <0.001, *η*^2^ = 0.036. However, significant differences between institutions were found with regard to this trend, *F*(5, 373) = 6.288, *P* <0.001, *η*^2^ = 0.078. A closer look within institutions reveals that this trend is statistically significant in HSPH, *F*(1, 108) = 31.738, *P* <0.001, *η*^2^ = 0.227; SPHFU, *F*(1, 59) = 4.230, *P* = 0.044, *η*^2^ = 0.067; and INSP, *F*(1, 60) = 18.465, *P* <0.001, *η*^2^ = 0.235; but not in SPHUWC or UMST. Finally, in KIT the trend appears to be reversed in that the difference is not statistically significant, *F*(1, 72) = 3.561, *P* = 0.063, *η*^2^ = 0.047.

## Discussion

Our study across six MPH programs is the first to ask graduates for attribution to the MPH program with regard to competencies and impact variables. Our study reports on one of the highest numbers of graduates of Masters in health and health care (n = 445); the highest was 478; response rates reported were similar [18].

The study shows that, after graduation, graduates worked less in clinics and district health departments and moved to international NGOs and research institutes. The change of work of graduates is similar to that reported by others [19,29,31]. Possibly, the high number of changes to “other” workplaces indicates a move to centers of disease control, which was not differentiated in the questionnaire.

In contrast to the reported brain-drain of higher educated health professionals [32-34], only 5% (24) of the graduates left their home country and, of these, seven left to work in a high income country; the remaining 17 left their country to work in their region of origin. Only five respondents, who had substantial experience in LMIC, came from high income countries.

Though traditionally medical doctors enrolled for an MPH, a wide range of different educational backgrounds are represented such as a Bachelor of Public Health, nursing, dentistry, and social sciences [35].

As for career and leadership, a large proportion of graduates changed their leadership position, technical position, acquired new responsibilities, and increased their remuneration, and attributed these changes to the MPH program. In other studies change in leadership, technical position or new responsibilities have been reported, however, in those studies it was not clear whether these changes occurred due to gaining seniority or other factors [19,21,29,34,36-41]. Richardson, in 2008, was the only one to ask about satisfaction with professional skills and professional status and the contribution of the program, in this case occupational therapy, to that satisfaction [20]. A higher salary was also reported by Bradley [42], Gill [39], Ruth [43], Drennan [41], although attribution was unclear.

An additional degree, as well as graduation from specific institutions, positively influenced change in leadership, technical position, and more responsibility; a medical degree positively influenced change in leadership. As for the institutions, having graduated from INSP, SPHUWC, or KIT seemed to be more beneficial to someone’s career in terms of change in leadership, technical position, and remuneration. Having graduated from INSP or KIT positively influenced being assigned more responsibility. As the contexts, countries, and programs are so different, it is difficult to surmise what might be the reason for the differences between the graduates of these institutions. In other studies, i.e., in the USA, gender influenced increase in remuneration; this was the case in our study as well [42].

About a third of the study participants undertook further studies other than short courses. A high proportion had completed their PhD (9%), this might result from the time lapse between graduation and the study; others indicated that they were in the process of studying towards a PhD [20,34,41,44]. Other studies also reported graduates undertaking further studies [20,21,37,41,42].

In relation to the application of competencies, almost half of the graduates stated that the MPH program contributed substantially to the application of public health competencies, though with large variations between institutions. Specifically, public health analytical competencies as well as leadership, and context-sensitive and planning and management competencies were mentioned. Other studies reported enhanced job skills and performance [45], a range of public health skills, or international health competencies [29,46]. A number of studies reported specific skills such as management [36,37]. A number of studies reported generic competencies such as critical reflection and critical thinking, which this study did not explore [21,47-53]. Policy development competencies were the least mentioned, which may arise from, for example, the place of work, the degree of emphasis by specific MPH programs, or the different processes of policy making.

As regards impact on the workplace, graduates attributed the enablement by the MPH program to impact on the workplace between 60% and 20% for specific impact variables, with a large difference between institutions. Importantly, graduates attributed, for example, “enablement in writing a research proposal” and “reporting/making recommendations on population health status”, as this is what would be expected from a public health professional [54,55]. The diverse range of areas of work (management, research, policy, teaching) might contribute to the fact that graduates reported, in a range of 38% to 14%, that each of the competencies was not used or not part of their work [56,57]. The highest reported variable not used or not part of their work was “contributed to writing a published chapter of a book”, which in hindsight might have been too high an expectation. Other studies seldom asked for impact on the workplace, or asked it only qualitatively, while only two studies reported quantitatively and/or attribution [20,22].

Concerning impact on society, graduates reported that the MPH program enabled them substantially to impact on society with variables rated from 39% to 17% for the 10 defined variables, with large ranges between institutions. “influencing better understanding of public health” and “contributing equitable access to quality services” are both important achievements in public health. Only two studies reported impact on society [20,22] and this study was the first which looked at defined impact. Though impact on society through higher educated professionals is very difficult to measure, because of many influencing factors before, during, and after the MPH program, the results give a good indication on what impact the graduates felt they did contribute.

### Limitations

Self-reporting measures are easy to administer simultaneously at different locations, they are relatively easy to subject to quantitative analysis, they are inexpensive, and are less time demanding than testing and assessment [24,26]. However, self-reported measures might be prone to biases such as an overly positive (i.e., loyalty of graduates to their MPH program) or exaggerated modesty, vagueness, and ambiguities of questions, and a tendency to give consistent evaluations across a set of specific items [24,26]. In this study the underlying factors for change in leadership and new responsibilities were not studied. It might be that those people who have potential to become leaders/managers have chosen the MPH as a relevant training program to prepare for a potential higher position. On the other hand, people trained as MPH may show to hold competencies that are necessary for a leadership position, or are supposed to have those competencies because of the degree, so they tend to be promoted. Next to self-reporting as such, the use of different “yardsticks” across programs and countries (anchoring), culture, or different programs having a different emphasis might result in bias [15,25,58]. However, the competencies and impact variables had been validated across countries before the study [28]. In order to reduce the risk of poor anchoring as well as the tendency to avoid extreme responses, the scales were kept as small as possible.

Using an online survey tool may have had some influence on completeness. Graduates may have left particular questions unanswered because of loss of connectivity with the institution or the online tool before or during the completion of the survey. Though the relatively low response rate influences the results of the study, as those who answered may have been more positive, the response rate of this study did not differ much from the response rate of other alumni surveys [28]. Efforts were exerted to find as many graduates as possible and to encourage them to fill in the survey.

The questionnaire could have been constructed differently by mixing items to reduce the halo error, however, that could have negatively impacted on the user-friendliness of the questionnaire. In order to increase validity, the questionnaire was pretested with graduates from all MPH programs and adjusted. The fact that the MPH program contributed less to the impact factors on society than the impact factors on the workplace can be seen as a measure of predictive validity as one would expect that graduates have less influence on society than on their workplace. As previously mentioned, other methods, such as semi-or unstructured interviews, could also have been used. This study is part of a larger research project, in which next to the alumni survey, 10 graduates per school, their peers, and their supervisors were interviewed. These results are being analyzed.

## Conclusions

This is the first transnational study on outcome and impact of MPH programs and the first transnational study on a Master’s in health and health care. From this study it can be concluded that, according to graduates, the MPH programs contribute to improvements in graduates’ careers and to leadership building in public health. The MPH programs contributed substantially to the application of public health analytical competencies as well as leadership, context-specific and planning and management competencies. Graduates reported substantial contribution by the MPH program on impact variables on the workplace such as development of a research proposal and reporting on population health needs. The contribution to impact variables on society, such as “contributing to equitable access to quality services”, was less. The differences between the MPH programs from different countries warrant further study in order to find explanations. It is argued that this study makes some progress in problematizing and measuring impact of MPH programs for the first time. It is also concluded that the follow-up of graduates, as performed in this study, is an efficient and practical way to reach a large number of respondents across countries and could be readily replicated. The results of the in-depth study are still to follow. Further strategies to enhance understanding of impact might be focus group discussions with graduates, though more costly and logistically difficult, or an employer survey. Finally, the findings could, and will be in the cases of the participating institutions, used to steer curriculum reform and innovation. For example “policy development competencies” were assessed as lowly attributed to the MPH program, and therefore curricula could include knowledge and skills building around analyzing, evaluating, and developing policy options for public health programs.

## Abbreviations

ANOVA: Analysis of variance; ANCOVA: Analysis of covariance; HSPH: Hanoi School of Public Health, Vietnam; INSP: National Institute of Public Health, Mexico; KIT: Royal Tropical Institute, Amsterdam, the Netherlands; MPH: Master of Public Health; LMIC: Low and middle income Countries; SPHUWC: School of Public Health, University of Western Cape, Cape Town, South Africa; SPHFU: School of Public Health, Fudan University, Shanghai, China; UMST: University of Medical Sciences and Technology, Khartoum, Sudan; WHO: World Health Organization.

## Competing interests

PZ is KIT’s MPH program director, and wrote this article during her sabbatical. Dr Hanan Tahir is the MPH program director at UMST.

## Authors’ contributions

PZ conceived the article. PZ, LA, NTH, QX, LMV, XHY, MCGR, MAW, and HT were all involved in enriching the original research idea and data collection. JL performed the initial statistical analysis. PZ wrote the first draft of the article. All authors contributed to data analysis, writing, and review. All authors read and approved the final manuscript.

## Supplementary Material

Additional file 1 Questionnaire as sent to graduates. Click here for file

Additional file 2: Table S4 Enablement of impact on work attributed to the MPH program as reported by graduates. Click here for file

Additional file 3: Table S5 Enablement of impact on society attributed to the MPH program as reported by graduates. Click here for file

Additional file 4: Tables S6–S10 Changes in position by predictor variables. Click here for file
