# Supplementary material for: Outcome and impact of Master of Public Health programs across six countries: education for change
Source: Hum Resour Health. 2014 Aug 6;12:40. doi: 10.1186/1478-4491-12-40 (PMC4130699; doi:10.1186/1478-4491-12-40)
Supplement: Additional file 1 — Questionnaire as sent to graduates. [file 1478-4491-12-40-S1.docx]

**ICHD/ Masters in Public Health Alumni Survey**

**18 December 2012**

**Dear graduate,**

Thank you very much for your consent to participate in this study of the application of Public Health competencies, as well as the possible outcome and impact of the Masters of Public Health program. The higher education institutions that run these programs are located across a range of countries and include: Fudan School of Public Health, Shanghai, China, Prof Qian Xu; Hanoi School of Public Health, Vietnam, Prof Nguyen Thi Huong, vice-dean National Institute of Public Health, Mexico, Dr Laura Magana Academic dean; University of the Western Cape, South Africa, Ms Lucy Alexander, Senior Academic Coordinator; Sudan through the Ministry of Health, Dr Nazar El Faki, Director Policy and Planning for Human resources for Health and the Royal Tropical Institute, Amsterdam, the Netherlands, Dr Prisca Zwanikken, Area Leader Education.

You will be asked questions about your work situation, the competencies that you applied, the changes that you possibly made in your workplace and the facilitating and hindering factors influencing this. The survey will take you approximately 25 minutes to fill in.

We will update you on the results of the survey through email.

Once again: thank you very much!

On behalf of the Research Team,

Rinia Sahebdin alumni officer

| **Number** | **Question** | **Answer** |
| --- | --- | --- |
| **0.** | I confirm that I understood the consent form and agree to participate in this survey. | Yes  No (please don’t proceed with the questions until you can state yes) |
| **1. DEMOGRAPHIC INFORMATION** | | |
| 1.1 | Sex | Female  Male |
| 1.2 | In which year were you born? | …. |
| 1.3 | Country of origin | ................................................................... |
| 1.4 | Country where you currently work | ................................................................... |
| 1.5 | What was your professional education, before you started the MPH program?  ***(Please select one option which is most appropriate to you)*** | Medical Doctor  Nurse/Midwife  Nurse  Pharmacist  Social Scientist  Dentist  Bachelor of Public Health  Other (please specify): ................................................................... |
| 1.6 | Did you study full time or part-time in the MPH program? | Full time  Part-time |
| 1.7 | In which year did you start your MPH? | ................................................................... |
| 1.8 | In which year did you complete your MPH? | ................................................................... |
| 1.9 | Number of years of work experience between your first degree and the start of your MPH program | ....................Years |
| 1.10 | What best describes your last place of employment BEFORE starting the MPH course? | National Ministry of Health  Regional/Provincial Health Department  District Health Department  Hospital or Clinic  College/University  Research Institute  International NGO  National NGO  Local NGO  Not employed  Other (please specify): ................................................................... |
| 1.11 | What best describes your main areas of work BEFORE starting the MPH.  ***(Please select a maximum of 3 options which are most appropriate to you)*** | Clinical care/service  Disease prevention or control  Rehabilitation  Health promotion (including health education, communication, social marketing, etc)  Teaching/training  Research  Information management  Public Health management  Program/project management  Public communication and involvement with stakeholders  Policy process involvement  Other (please specify): ................................................................... |
| **2. CURRENT EMPLOYMENT** | | |
| 2.1 | Number of years of public health related work experience since your MPH graduation | .......... years |
| 2.2 | After graduating from the MPH, did you change your leadership level in the management system? | No → Please go to Question 2.4  Yes → Please go to Question 2.3 |
| 2.3 | Please rate the contribution of the MPH graduation to your change of leadership level:  ***(Please select ONE number that most applies to your case)***  1 2 3 4 5  Insignificant Little Significance Moderate Significance Significant Very significant | |
| 2.4 | After graduating from the MPH, did you change your technical position or area of focus(Your function but at the same level)? | Yes → Please go to Question 2.5  No → Please go to Question 2.6 |
| 2.5 | Please rate the contribution of the MPH graduation to your change in technical position or area of focus.  ***(Please select ONE number that most applies to your case)***  1 2 3 4 5  Insignificant Little Significance Moderate Significance Significant Very significant | |
| 2.6 | Did you acquire any new responsibilities after graduating with the MPH? | No, it remained the same  Yes |
| 2.7 | After graduating from the MPH, did your work remuneration change compared to your work remuneration BEFORE starting the MPH?  ***(Please select one option which is most appropriate to you)*** | Current work remuneration is lower than before graduating from the MPH. Please move to Question 2..8  Current work remuneration is the same as before graduating from the MPH. Please go to Question 2.8  Current work remuneration is higher since graduating from the MPH. Please go to Question 2.8 |
| 2.8 | Please rate the contribution of MPH graduation to your change in remuneration [the salary change or lack of change]:  ***(Please select ONE number that most applies to your case)***  1 2 3 4 5  Insignificant Little Significance Moderate Significance Significant Very significant | |
| 2.9 | After completing the MPH did you move to a different employer/organisation? | Yes → Please go to Question 2.10  No → Please go to Question 2.11 |
| 2.10 | Please rate the contribution of MPH graduation of you going to a different employer/organisation:  ***(Please select ONE number that most applies to your case)***  1 2 3 4 5  Insignificant Little Significance Moderate Significance Significant Very significant | |
| 2.11 | What best describes your CURRENT place of employment?  ***(Please select ONE option which is most appropriate to you)*** | National Ministry of Health  Regional/Provincial Health Department  District Health Department  Hospital or Clinic  College/University  Research institute  International NGO  National NGO  Local NGO  Not employed  Other (please specify): ................................................................... |
| 2.12 | What are your main areas of work which describe what you are doing IN YOUR CURRENT WORKPLACE.  ***(Please select a maximum of THREE options which are most appropriate to you)*** | Clinical care/clinical service  Disease prevention or control  Rehabilitation  Health promotion (including health education, communication, social marketing, etc)  Teaching/training  Research  Information management  Public Health management  Program/project management  Public communication and involvement with stakeholders  Policy process involvement  Other (please specify): ................................................................... |
| **3. WORK-RELATED TRAINING** | | |
| 3.1 | Since graduating from the MPH, have you completed any additional certificated work-related training for TWO weeks or longer? | No  Yes. Please specify the number of training courses attended …………………… |
| 3.2 | Have you embarked on any further degree or diploma studies (qualifications other than short training courses)? | No → Please go to Question 3.4  Yes → Please go to Question 3.3 |
| 3.3 | Please mark as many degrees/diplomas as you have been awarded after completing your MPH | Diploma  Postgraduate Diploma  Masters degree  PhD  Post doctorate award  Other (Please specify):...................... |
| 3.4 | Do you plan to embark on any further studies for a degree/diploma (qualifications other than short training courses)? | No  Yes |

**4. Please rate to what extent the MPH program enabled you to apply specific public health competencies in your work?**

# TABLE A: KEY COMPETENCIES ANTICIPATED IN A MPH GRADUATE

| **CATEGORY OF COMPETENCY** | DETAILED COMPETENCIES | ***Please select ONE number for each sub-question***  ***that most applies to your case.***  **The MPH degree has enabled me to apply this competency:**  **1.No- I don’t use it/ It is not part of my work**  **2.Not due to the MPH**  **3.MPH enabled me a little to apply this competency**  **4.MPH enabled me substantially to apply this competency** |
| --- | --- | --- |
| 1. Public Health science skills including analytical assessment competencies | 1. Applies the basic Public Health sciences (including but not limited to biostatistics, epidemiology, environmental health services, health services administration and social and behavioral health sciences) to Public Health policies and programs | 1 2 3 4 |
|  | 2. Appraises scope, function and role of Public Health in relation to local context, health system and other social sectors. | 1 2 3 4 |
|  | 3. Assesses population health status and identifies population health problems, risk factors, related Social Determinants, and determines needs. | 1 2 3 4 |
|  | 4. Commissions and critically interprets research findings and/or develops protocol and collects, analyses and synthesizes reliable and valid data using qualitative and quantitative methods. | 1 2 3 4 |
| 2.Policy process competencies | 5. Analyzes and evaluates policy options and determines feasibility for Public Health policies/ programs in diverse community contexts, using appraisal of evidence. | 1 2 3 4 |
|  | 6. Participates in developing context sensitive policies and strategic plans and translates them into action. | 1 2 3 4 |
|  | 7. Understands and contributes to developing and using mechanisms to monitor and evaluate Public Health policies and regulations. | 1 2 3 4 |
|  | 8. Contributes to advocacy of new and existing health policies to the public health and other sectors. | 1 2 3 4 |
| 3. Communication competencies | 9. Communicates concisely in writing and orally, in person and through electronic means with linguistic and cultural proficiency and appropriateness. | 1 2 3 4 |
|  | 10. Facilitates and integrates input to Public Health policy and programs from a wide range of individual and organizational stakeholders. | 1 2 3 4 |
|  | 11. Uses a variety of culturally appropriate approaches to disseminate Public Health information with consideration to ethical and confidential issues. | 1 2 3 4 |
| 4. Context sensitive  Competencies | 12. Analyzes the role of gender, cultural, social, economic, political and behavioral factors in the accessibility, availability, acceptability and delivery of Public Health services and programs. | 1 2 3 4 |
|  | 13. Incorporates a Social Determinants of Health approach to Public Health needs. | 1 2 3 4 |
| 5. Community and inter-sectoral competencies | 14. Assesses and engages community actors and communities and their linkages and relationships that affect health in diverse social and cultural situations. | 1 2 3 4 |
|  | 15. Collaborates in community-based participatory efforts. | 1 2 3 4 |
|  | 16. Develops and maintains partnerships with key stakeholders, including from different sectors. | 1 2 3 4 |
| 6. Planning and management competencies | 17. Uses evidence and good practice to address Public Health policy, planning and management issues. | 1 2 3 4 |
|  | 18. Plans, implements, monitors and evaluates Public Health interventions, programs, resources, services including input, process, outcome and impact. | 1 2 3 4 |
|  | 19. Prepares and contributes to manage and evaluate Public Health information systems, human, financial and logistic resources. | 1 2 34 |
| 7. Leadership and systems thinking competencies | 20. Demonstrates leadership as a manager and in team efforts, and is able to lead in Public Health emergencies. | 1 2 34 |
|  | 21. Demonstrates professional judgment and ethical standards in data handling and addressing Public Health issues and diverse opinions. | 1 2 34 |
|  | 22. Leads with applying the understanding of the interconnectedness and dynamic interactions of the Public Health system. | 1 2 34 |
|  | 23. Continues life-long learning and professional development, and stimulates team to do so. | 1 2 34 |

| 4.2 Please rate the effects of the following factors on applying the competencies gained during your MPH. Please use the following scale:  -1 0 1  Hindered Did not hinder Facilitated | | | | |
| --- | --- | --- | --- | --- |
| **Factors** | | **Influences *(Please select ONE rating for each sub-question that most applies to your case)*** | | |
|  |  | **Hindered** | **Did not hinder** | **Facilitated** |
| 1 | Change/s of job | **-1** | **0** | **1** |
| 2 | Workload | **-1** | **0** | **1** |
| 3 | Relationship with peers | **-1** | **0** | **1** |
| 4 | Relationship with line managers | **-1** | **0** | **1** |
| 5 | Organizational culture | **-1** | **0** | **1** |
| 6 | Gender issues | **-1** | **0** | **1** |
| 7 | Workplace learning | **-1** | **0** | **1** |
| 8 | Workplace policies/policies regulating the system | **-1** | **0** | **1** |
| 9 | Political situation | **-1** | **0** | **1** |
| 10 | Labor market issues | **-1** | **0** | **1** |
| 11 | Family responsibilities/ Family related event | **-1** | **0** | **1** |
| 12 | Others. Please specify……………………. | **-1** | **0** | **1** |

5. **Please rate to what extent the MPH program enabled you to impact on your workplace?**

Please note that, depending on your work or the context of your work or workplace, it is possible that not all variables apply to you.

| **TABLE B:** **ANTICIPATED** **IMPACT VARIABLES AT THE WORKPLACE** | **Please rate for each sub-question to what extent the MPH program enabled you to impact on your workplace**  1. Not applicable to me/ my workplace  2. My impact is not attributable to the MPH.  3. The MPH enabled me a little to impact on my workplace.  4.The MPH enabled me substantially to impact on my workplace |
| --- | --- |
| 1. Created evidence (primary or secondary) for decision-making. | 1 2 3 4 |
| 2. Developed a study or a research proposal. | 1 2 3 4 |
| 3. Reported and made recommendations on population health status or needs. | 1 2 3 4 |
| 4. Contributed to change in policy at workplace where needed. | 1 2 3 4 |
| 5. Contributed to change in policy at one level higher than work institution. | 1 2 3 4 |
| 6. Participated and influenced working committees for program design or policy formulation at provincial, national or international level. | 1 2 3 4 |
| 7. Published or posted in popular (including electronic) media. | 1 2 3 4 |
| 8. Made presentations at conferences. | 1 2 3 4 |
| 9. Published in peer reviewed publications. | 1 2 3 4 |
| 10. Contributed to writing a published chapter of a book. | 1 2 3 4 |
| 11. Tutored or taught Public Health professionals, trainees or students in the community. | 1 2 3 4 |
| 12. Developed, reviewed or commissioned educational or Health Promotion media and materials. | 1 2 3 4 |
| 13. Planned or implemented community health education courses and workshops. | 1 2 3 4 |
| 14. Intervened or worked with a Social Determinants of Health Framework in a way that promotes equity and/or is pro-poor. | 1 2 3 4 |
| 15. Collaborated/networked/developed partnerships successfully with other departments than health. | 1 2 3 4 |
| 16. Initiated, sustained and evaluated projects with community participation. | 1 2 3 4 |
| 17. Planned and implemented Public Health interventions, programs or policies based on consultation with stakeholders and using evidence and best practice. | 1 2 3 4 |
| 18. Implemented performance improvement strategies in response to monitoring and evaluation findings. | 1 2 3 4 |
| 19. Contributed to improvements in human resource management. | 1 2 3 4 |
| 20. Contributed to improving regular working procedures. | 1 2 3 4 |
| 21. Instrumental in initiating a change within the workplace, or at some level beyond. | 1 2 3 4 |
| 22. Contributed to addressing the determinants of health e.g. through planning processes, resource allocation or research. | 1 2 3 4 |
| 23. Raised a project grant. | 1 2 3 4 |
| 24. Contributed to reputation-building of workplace. | 1 2 3 4 |
| 25. Participated in national and international collaboration. | 1 2 3 4 |
| 26. Participated in building a successful partnership. | 1 2 3 4 |

6. **Please rate to what extend the MPH program enabled you to impact on society.**

Please note that, depending on your work or the context of your work, workplace or society, it is possible that not all variables apply to you.

| **TABLE C: IMPACT VARIABLES ON SOCIETY** | **Please rate for each sub-question to what extend the MPH degree has enabled you to impact on society**  1. Not applicable to me  2. My impact is not attributable to the MPH.  3. The MPH enabled me a little to impact on society  4. The MPH enabled me substantially to impact on society |
| --- | --- |
| 1. Contributed to changes in policy or strategy in general. | 1 2 3 4 |
| 2. Contributed to changed guidelines, regulations, ordinances beyond the workplace. | 1 2 3 4 |
| 3. Contributed to influencing communities, organisations, health sector and other sectors than health. | 1 2 3 4 |
| 4. Contributed to equity/pro-poor orientation towards health access at all levels. | 1 2 3 4 |
| 5. Contributed to changes in resource allocation for interventions, and research, orientated towards equity and addressing the determinants of health. | 1 2 3 4 |
| 6. Contributed to equitable access to quality services. | 1 2 3 4 |
| 7. Contributed to improved Public Health in specific areas related to work context, e.g. improved utilization of services. | 1 2 3 4 |
| 8. Contributed to increased resource mobilization for Public Health. | 1 2 3 4 |
| 9. Contributed to increased resource mobilization for disadvantaged groups. | 1 2 3 4 |
| 10. Influenced better understanding of Public Health measures amongst general population. | 1 2 3 4 |

Once again: thank you very much for your time!
