# Supplementary material for: Outcome and impact of Master of Public Health programs across six countries: education for change
Source: Hum Resour Health. 2014 Aug 6;12:40. doi: 10.1186/1478-4491-12-40 (PMC4130699; doi:10.1186/1478-4491-12-40)
Supplement: Additional file 2: Table S4 — Enablement of impact on work attributed to the MPH program as reported by graduates. [file 1478-4491-12-40-S2.docx]

**Additional file 2**

Table S4: The extent to which the MPH program enabled the graduate to impact on the workplace (*N*=418)*

| **Impact variables at workplace / attribution to MPH program** | MPH enabled me substantially to impact on my workplace  (%) | MPH enabled me a little to impact on my workplace  (%) | Not due to MPH  (%) | Not use/ not part of my work (%) |
| --- | --- | --- | --- | --- |
| 1. Created evidence (primary or secondary) for decision-making. | 37.5 | 32.1 | 5.7 | 24.7 |
| 2. Developed a study or a research proposal. | 60.4 | 27.7 | 4.3 | 7.6 |
| 3. Reported and made recommendations on population health status or needs. | 43.3 | 35.7 | 6.2 | 14.8 |
| 4. Contributed to change in policy at workplace where needed. | 31.0 | 39.0 | 11.3 | 18.7 |
| 5. Contributed to change in policy at one level higher than work institution. | 22.3 | 50.2 | 15.7 | 28.5 |
| 6. Participated and influenced working committees for program design or policy formulation at provincial, national or international level. | 27.3 | 35.8 | 9.5 | 27.3 |
| 7. Published or posted in popular (including electronic) media. | 23.3 | 31.1 | 16.4 | 29.2 |
| 8. Made presentations at conferences. | 41.0 | 31.0 | 14.6 | 13.4 |
| 9. Published in peer reviewed publications. | 26.5 | 27.6 | 13.5 | 32.5 |
| 10. Contributed to writing a published chapter of a book. | 19.8 | 26.5 | 15.7 | 38.0 |
| 11. Tutored or taught public health professionals, trainees or students in the community. | 36.6 | 30.6 | 10.3 | 22.5 |
| 12. Developed, reviewed or commissioned educational or Health Promotion media and materials. | 28.7 | 31.5 | 11.8 | 28 |
| 13. Planned or implemented community health education courses and workshops. | 38.5 | 33 | 8.5 | 20.0 |
| 14. Intervened or worked with a Social Determinants of Health Framework in a way that promotes equity and/or is pro-poor. | 32.6 | 29.8 | 9.3 | 28.3 |
| 15. Collaborated/networked/developed partnerships successfully with other departments than health. | 34.7 | 34 | 11.8 | 19.5 |
| 16. Initiated, sustained and evaluated projects with community participation. | 29.3 | 32.8 | 11.9 | 26 |
| 17. Planned and implemented Public Health interventions, programs or policies based on consultation with stakeholders and using evidence and best practice. | 35.6 | 35.3 | 10 | 19.1 |
| 18. Implemented performance improvement strategies in response to monitoring and evaluation findings. | 32.5 | 38.5 | 10.7 | 18.3 |
| 19. Contributed to improvements in human resource management. | 30.3 | 33.4 | 13.1 | 23.2 |
| 20. Contributed to improving regular working procedures. | 32.4 | 37.5 | 14.7 | 15.4 |
| 21. Instrumental in initiating a change within the workplace, or at some level beyond. | 27.5 | 37.4 | 15 | 20.1 |
| 22. Contributed to addressing the determinants of health e.g. through planning processes, resource allocation or research. | 35.2 | 36.1 | 11.4 | 17.3 |
| 23. Raised a project grant. | 24.1 | 31.5 | 12.4 | 32 |
| 24. Contributed to reputation-building of workplace. | 30 | 36.2 | 13.1 | 20.7 |
| 25. Participated in national and international collaboration. | 34.4 | 30.9 | 11.5 | 23.2 |
| 26. Participated in building a successful partnership (added) | 37.3 | 35.9 | 12.7 | 14.1 |

*Missing: 27
