# Supplementary material for: Outcome and impact of Master of Public Health programs across six countries: education for change
Source: Hum Resour Health. 2014 Aug 6;12:40. doi: 10.1186/1478-4491-12-40 (PMC4130699; doi:10.1186/1478-4491-12-40)
Supplement: Additional file 3: Table S5 — Enablement of impact on society attributed to the MPH program as reported by graduates. [file 1478-4491-12-40-S3.docx]

**Additional file 3**

Table S5:The extent to which the MPH program enabled the graduate to impact on society (*N*=418)*

| **Impact variables on society/ attribution to MPH program** | MPH enabled me substantially to impact on society  (%) | MPH enabled me a little to impact on society  (%) | Not due to MPH  (%) | Not use/ not part of my work  (%) |
| --- | --- | --- | --- | --- |
| 1. Contributed to changes in policy or strategy in general | 24.9 | 30.2 | 15.6 | 29.3 |
| 2. Contributed to changed guidelines, regulations, ordinances beyond the workplace. | 17 | 38.8 | 14.5 | 29.7 |
| 3. Contributed to influencing communities, organisations, health sector and other sectors than health. | 25.2 | 36.9 | 14.9 | 23 |
| 4. Contributed to equity/pro-poor orientation towards health access at all levels. | 24.6 | 35.7 | 12.4 | 27.3 |
| 5. Contributed to changes in resource allocation for interventions, and research, orientated towards equity and addressing the determinants of health. | 25.7 | 31.8 | 14.4 | 28.1 |
| 6. Contributed to equitable access to quality services. | 31.7 | 34.6 | 11 | 22.7 |
| 7. Contributed to improved Public Health in specific areas related to work context, e.g. improved utilization of services. | 30.2 | 36.7 | 12.7 | 20.4 |
| 8. Contributed to increased resource mobilization for Public Health. | 27.2 | 29.1 | 14.3 | 29.4 |
| 9. Contributed to increased resource mobilization for disadvantaged groups. | 31.1 | 30.4 | 13.9 | 24.7 |
| 10. Influenced better understanding of Public Health measures amongst general population. | 39.3 | 36.5 | 7.1 | 17.1 |

*Missing: 27
