# Supplementary material for: Outcome and impact of Master of Public Health programs across six countries: education for change
Source: Hum Resour Health. 2014 Aug 6;12:40. doi: 10.1186/1478-4491-12-40 (PMC4130699; doi:10.1186/1478-4491-12-40)
Supplement: Additional file 4: Tables S6–S10 — Changes in position by predictor variables. [file 1478-4491-12-40-S4.docx]

**Additional file 4**

**Tables S6–S10: Changes in position by predictor variables, logistic regression**

Tables S6-S10 present the outcomes of logistic regression for *change in leadership position (yes/no), switch in technical position (yes/no), switch to position involving more responsibility (yes/no), increase in remuneration (yes/no), change in employer (yes/no).*

Table S6

Logistic regression for switch to leadership position (0=no, 1=yes): regression coefficients (*B*) along with standard errors (*SE*), *p*-values, Odds Ratios (*OR*) and confidence intervals (CI).

Term *B* (*SE*) *p*-value *OR* 95% CI for *OR*

Lower Upper

Intercept 1.076 (0.216) < 0.001

Medical doctor ^1^ 0.801 (0.247) 0.001 2.229 1.373 3.617

Additional degree ^1^ 0.711 (0.253) 0.005 2.035 1.239 3.342

HSPH ^2^ -1.455 (0.273) < 0.001 0.233 0.137 0.399

SPHUWC ^2^ -0.894 (0.372) 0.016 0.409 0.197 0.848

UMST ^2^ -2.049 (0.419) < 0.001 0.129 0.057 0.293

^1^ no=0, yes=1; ^2^ compared to SPHFU, INSP and KIT which did not differ significantly from each other

Table S7

Logistic regression for switch to technical position (0=no, 1=yes): regression coefficients (*B*) along with standard errors (*SE*), *p*-values, Odds Ratios (*OR*) and confidence intervals (CI).

Term *B* (*SE*) *p*-value *OR* 95% CI for *OR*

Lower Upper

Intercept 1.391 (0.208) < 0.001

Additional degree ^1^ 0.575 (0.248) 0.021 1.776 1.092 2.889

HSPH ^2^ -1.311 (0.255) < 0.001 0.270 0.164 0.444

SPHFU ^2^ -1.028 (0.339) 0.002 0.358 0.184 0.695

UMST ^2^ -1.263 (0.401) 0.002 0.283 0.129 0.621

^1^ no=0, yes=1; ^2^ compared to SPHUWC, INSP and KIT which did not differ significantly from each other

Table S8

Logistic regression for switch to position involving more responsibility (0=no, 1=yes): regression coefficients (*B*) along with standard errors (*SE*), *p*-values, Odds Ratios (*OR*) and confidence intervals (CI).

Term *B* (*SE*) *p*-value *OR* 95% CI for *OR*

Lower Upper

Intercept 3.033 (0.461) < 0.001

Additional degree ^1^ 1.141 (0.343) 0.001 3.131 1.597 6.136

HSPH ^2^ -1.971 (0.502) < 0.001 0.139 0.052 0.372

SPHFU ^2^ -2.967 (0.528) < 0.001 0.051 0.018 0.145

SPHUWC ^2^ -2.366 (0.573) < 0.001 0.094 0.031 0.288

UMST ^2^ -2.706 (0.594) < 0.001 0.067 0.021 0.214

^1^ no=0, yes=1; ^2^ compared to INSP and KIT which did not differ significantly from each other

Table S9

Logistic regression for increase in remuneration (0=no, 1=yes): regression coefficients (*B*) along with standard errors (*SE*), *p*-values, Odds Ratios (*OR*) and confidence intervals (CI).

Term *B* (*SE*) *p*-value *OR* 95% CI for *OR*

Lower Upper

Intercept 1.276 (0.243) < 0.001

Time of graduation ^1^ -0.636 (0.226) 0.005 0.529 0.340 0.825

HSPH ^2^ -0.954 (0.252) < 0.001 0.385 0.235 0.631

SPHFU ^2^ -0.700 (0.335) 0.037 0.496 0.257 0.958

UMST ^2^ -1.123 (0.392) 0.004 0.325 0.151 0.702

Gender ^3^ 0.608 (0.217) 0.005 1.836 1.199 2.811

^1^ 2005-2007=0, 2008-2010=1; ^2^ compared to SPHUWC, INSP and KIT which did not differ significantly from each other; ^3^ woman=0, man=1

Table S10

Logistic regression for switch to another employer (0=no, 1=yes): regression coefficients (*B*) along with standard errors (*SE*), *p*-values, Odds Ratios (*OR*) and confidence intervals (CI).

Term *B* (*SE*) *p*-value *OR* 95% CI for *OR*

Lower Upper

Intercept 0.468 (0.181) 0.010

Time of graduation ^1^ -0.722 (0.202) < 0.001 0.486 0.327 0.722

HSPH ^2^ -0.465 (0.217) 0.032 0.628 0.411 0.961

SPHFU ^2^ -1.042 (0.331) 0.002 0.353 0.327 0.722

^1^ 2005-2007=0, 2008-2010=1; ^2^ compared to SPHUWC, INSP, UMST and KIT which did not differ significantly from each other
